# Kidney Stones in Primary Hyperoxaluria: New Lessons Learnt

**DOI:** 10.1371/journal.pone.0070617

**Published:** 2013-08-05

**Authors:** Dorrit E. Jacob, Bernd Grohe, Michaela Geßner, Bodo B. Beck, Bernd Hoppe

**Affiliations:** 1 Department of Geosciences, Johannes Gutenberg University Mainz, Mainz, Germany; 2 Schulich School of Medicine and Dentistry, School of Dentistry, Western University, London, Canada; 3 Division of Pediatric Nephrology, Department of Pediatric and Adolescent Medicine, University Hospital Cologne, Cologne, Germany; 4 Institute of Human Genetics, University of Cologne, Cologne, Germany; 5 Division of Pediatric Nephrology & German Hyperoxaluria Center, Department of Pediatrics, University Hospital Bonn, Bonn, Germany; UCL Institute of Child Health, United Kingdom

## Abstract

To investigate potential differences in stone composition with regard to the type of Primary Hyperoxaluria (PH), and in relation to the patient’s medical therapy (treatment naïve patients versus those on preventive medication) we examined twelve kidney stones from ten PH I and six stones from four PH III patients. Unfortunately, no PH II stones were available for analysis. The study on this set of stones indicates a more diverse composition of PH stones than previously reported and a potential dynamic response of morphology and composition of calculi to treatment with crystallization inhibitors (citrate, magnesium) in PH I. Stones formed by PH I patients under treatment are more compact and consist predominantly of calcium-oxalate monohydrate (COM, whewellite), while calcium-oxalate dihydrate (COD, weddellite) is only rarely present. In contrast, the single stone available from a treatment naïve PH I patient as well as stones from PH III patients prior to and under treatment with alkali citrate contained a wide size range of aggregated COD crystals. No significant effects of the treatment were noted in PH III stones. In disagreement with findings from previous studies, stones from patients with primary hyperoxaluria did not exclusively consist of COM. Progressive replacement of COD by small COM crystals could be caused by prolonged stone growth and residence times in the urinary tract, eventually resulting in complete replacement of calcium-oxalate dihydrate by the monohydrate form. The noted difference to the naïve PH I stone may reflect a reduced growth rate in response to treatment. This pilot study highlights the importance of detailed stone diagnostics and could be of therapeutic relevance in calcium-oxalates urolithiasis, provided that the effects of treatment can be reproduced in subsequent larger studies.

## Introduction

The primary hyperoxalurias (PH I, II and III) are rare, but underdiagnosed autosomal-recessively inherited disorders of the glyoxylate metabolism [1], [2]. Recurring urolithiasis and/or progressive nephrocalcinosis (the latter not yet observed in PH III) usually occurring early in childhood are their clinical hallmarks [1]–[4].

Among the three subtypes PH I is the most prevalent and most disastrous form [1]–[5]. Although rare (estimated prevalence rate <3 per 10^6^ population [6]–[12] with higher rates reported from some inbred populations [13], [14]). PH I regularly causes end-stage renal disease (ESRD) and curative treatment requires combined liver kidney and/or pre-emptive liver transplantation [15]. Even in industrialized countries there is a high rate of late diagnosis in advanced renal failure or after kidney graft failure in the setting of isolated kidney transplantation (up to 40% in adults), which denotes underreporting [6]–[10]. Infantile oxalosis occurring with generalized nephrocalcinosis and ESRD within the first 3 years of life constitutes the most severe PH I subgroup (up to 20% of cases) and still poses a major therapeutic challenge [1], [2], [16].

Despite marked hyperoxaluria in the primary range no case of ESRD has been reported yet for PH III [3]–[5]. It is still unclear, why there is such rate of high clinical remission over time, although hyperoxaluria and hypercalciuria seem to persist.

Current conservative management in PH is mainly identical to that in idiopathic calcium-oxalate (CaOx) stone disease and is based on a high fluid intake (>2 l per m^2^ body surface area per day) combined with medication to increase the urinary solubility index (alkaline citrate, orthophosphate and/or magnesium [1], [2], [15], [17]). The only specific drug available to PH I patients is pyridoxine (vitamin B6), the natural cofactor of the defective alanine-glyoxylate aminotransferase enzyme (*AGT*). Given in supraphysiological doses, vitamin B6, a pharmacoperone for susceptible *AGXT* missense mutations (e.g. c.508G>A), acts in multiple ways [18], finally leading to a reduced rate of endogenous oxalate generation and decreased urinary oxalate excretion [19], [20]. In the long run vitamin B6 treatment, even in (potentially) susceptible PH I genotypes, does not seems sufficient to overcome the deterious effects of massive hyperoxaluria [1], [15], [20], [21].

Early definite diagnosis of primary hyperoxaluria, especially for type I, is of utmost importance and mainly relies on genetic testing in industrialized countries today [22]. As the majority of PH patients present with urolithiasis and their stones have been reported to be distinctly different from idiopathic calcium-oxalate (CaOx) stones [23], [24], a concrement-based diagnostic approach could be extremely useful as a general screening tool in urolithiasis, particularly in countries with lower economic resources.

Based on descriptions of stones from PH patients by Daudon et al. [23], [24], who reported numerous differences between idiopathic CaOx stones and those of PH patients (mainly PH I), we are exploring here stone characteristics in relation to the precise PH type and genotype in greater detail using light and scanning electron microscopy as well as Micro-Raman spectrometry on surfaces and polished cross-sections. Twelve stones from ten patients diagnosed with PH I and six stones from four patients diagnosed with PH III could be obtained for the study (Table 1). Unfortunately, no stones form PH type II (GRHPR deficiency), the least common PH subtype in Germany [4] were available for analysis. In addition, we tried to differentiate between stones from naïve newly-diagnosed and therefore untreated patients (one patient diagnosed with PH I (1 stone) and one with a PH III diagnosis (2 stones, marked with an asterisk in Table 1) and stones formed under treatment with citrate and/or vitamin B6.

**Table 1:** Synopsis of stones used in this study with details of preventive treatment and genotype.

Sample	PHsubtype	Gender,age (yr)	StoneComposition^1^	Age at firstsymptom/ageat diagnosis	Past/*Current* clinicalsymptoms	Urinary Calciumexcretion (mg/kg/d)Diagnosis/current	Urinary oxalateexcretion(mmol/1.73 m^2^/d)Diagnosis/current	Plasmaoxalate(µmol/l)current	Treatment attime of stoneextraction,length (yrs)	Genotype
H1	PH I	M, 21	COM	2/5	Recurrent UL/*None*	1.04/3.05	2.56/0.63	13.24	Citrate+ Vit B6(p.resp.) (16)	c.508G>A/c.958delCA
H2	PH I	M, 15	COM	1/7	Recurrent UL/*None*	4.83/0.83	2.32/1.37	8.43	Citrate+ Vit B6(responsive) (9)	c.454T>A/c.1151T>C
H3, H5	PH I	M, 21	COM, COM	4/12	Recurrent UL/*Post staghorn* *calculus, mal-compliance*	1.6/1.09	1.08/0.92	16.69	Citrate+ Vit B6(responsive) (9)	c.508G>A/c.846+1G>T
H6	PH I	M, 12	COM	3/5	Recurrent UL/*Post transureteral* *stone removal, intermittent* *unexpl. hypercalciuria*	0.95/5.05	1.61/2.46	10.68	Citrate+ Vit B6 (9)(HCT since 01/12)	c.481G>A/c.976DelG
H9	PH I	F, 18	COM	3/12	Recurrent UL/*None*	1.8/1.75	1.76/1.48	11.14	Citrate+ Vit B6(responsive) (8)	c.508G>A/c.846-3C>G
H10	PH I	M, 25	COM	?/20 (ESRD)	Recurrent UL, mal-compliance/*Post combined liver-kidney Tx*	2.12 (post Tx)	5.32 (pre Tx) 0.37(post Tx)	7.48	Citrate (post Tx 7years) Vit B6(acute post Txperiod, respons.)	c.508G>A/c.846-3C>G
H18*	PH I	M, 23	COD+COM	4/14	Recurrent UL/*Acute stone* *passage in 06/12*	n.d./2.95	1.61/2.14	16.8	None, (9)	c.121G>A/C.846+1G>A
H21b, H21w	PH I	M, 44	COD+COM+CAP;COM, CAP	3/36	Recurrent UL/*Systemic Oxalosis,* *bones, GFR ∼ 40 ml/min*	0.6/0.35	2.76/2.92	35.26	Citrate+ Mg (8)	c.449C>T/c.1110del
H25	PH I	F, 16	COM	2/6	Recurrent UL/*Last* *stone episode 05/11*	0.75/1.19	3.16/1.87	14.46	Citrate+ Vit B6(not respons.) (10)	c.847-1G>C homozygous
H27	PH I	F, 7	COM	1/3	NC Grade I/*NC Grade*	n.d./0.74	1.7/0.61	19.64	Citrate+ Vit B6(respons.) (4)	c.508G>A homozygous
H4	PH III	F, 14	COD	1/13	Recurrent UL infirst decade of life/*None*	2.29/1.35	1.95/0.88	10.84	Citrate (12)	c.221T>G/c.700+5G>T
H7*, H13,H24	PH III	M, 3	H7: COD+COM,H13: COD+COM,H24: CAP+COD	3 months/8months	Recurrent UL in first yearsof life/*Stones in situ,* *no current stone passage*	Ca/Crea ratios0.5–0.8 mol/mol	0.8 Ox/Crea ratioup to 3 mol/mol	17.62	None at H7,Citrate (2)	c.728C>A homozygous
H11	PH III	M, 5	COD+COM	1/1.5	Recurrent UL and stoneremoval duringfirst year of life/*Stones in situ,* *no current stone passage*	Ca/Crea0.42 mol/mol/4.46	Ox/Crea1.51 mol/mol/2.87	12.26	Citrate (3)	c.221T>G/c.700+5G>T
H28*	PH III	M, 3	CAP+COM+COD	1.5/1.5	UL, stone removalprocedure/*None*	Ca/Crea0.36/0.78 mol/mol	Ox/Crearatio 0.449/0.316 mol/mol	n.d.	None (1.5)	c.700+5G>T homozygous

Asterisk denotes stones from newly-diagnosed and at the time untreated patients.

1 stone composition determined by multiple spot analyses on one polished surface. UL = Urolithiasis, NC = Nephrocalcinosis, HCT = Hydrochlorothiazide, GFR = Glomerular Filtration Rate, p.resp. = partly responsive to vit. B6 treatment.

## Materials and Methods

### Ethics Statement

The ethics statement for this study is IRB vote 06-231 (“Genotype-phenotype correlation for patients with primary hyperoxaluria”) obtained through the Ethics Commission of the University Hospital Cologne. The ethics committee specifically approved this study. All patients gave their written informed consent to the study and the ethics committee approved of this procedure.

### Patients

In all patients repeated 24 h urine analysis showed severe hyperoxaluria in the primary range (>1.0 mmol/1.73****m^2^/d), together with an elevated excretion of glycolate (PH I), or calcium (PH III). Diagnosis of PH I and PH III was established by complete PH I-III (*AGXT*, *GRHPR* and *HOGA1*) sequencing in all study participants and subsequent identification of two causative *AGXT* (PH I), respectively *HOGA 1* (PH III) mutations (Table 1).

### Sample Preparation and Light-Microscopy

Images of stone fragments were taken using a MZ125 binocular microscope (Leica, Wetzlar, Germany, 40× magnification) equipped with a Canon digital camera. Selected fragments were embedded in epoxy resin (Struers, Willich, Germany) then polished in several steps using 800 and 1200 grit Al_2_O_3_ powder followed by a last polishing step with 1 µm Al_2_O_3_ powder on a Buehler G-cloth. Light microscopy investigations and digital imaging of the polished surfaces were carried out with a VHX-600 digital microscope (KEYENCE, Neu-Isenburg; Germany), equipped with a VH-Z25 zoom lens (magnification from 25× to 175×) using polarized light.

### Scanning Electron Microscopy (SEM), Energy Dispersive X-ray Analysis (EDX)

A LEO (1540XB, Carl Zeiss, Jena, Germany) scanning electron microscope equipped with a Gemini field emission column was employed to determine compactness of stone masses, surface structure and habit (shape, dimensions, assembly) of formed aggregates and crystal phases and the surface structure of stone matrices. Stone fragments were coated with osmium (∼ 4 nm thickness; Plasma Coater OPC-80T) and investigated using an acceleration voltage of 1 kV and a working distance between 3 and 5 mm. The chemical composition of stones was analyzed in several different spots of each sample using EDX (Oxford INCA Instruments) at an acceleration voltage of 20 kV (penetration depth ∼2 µm). At least three randomly chosen spots in surface regions and in regions within a stone fragment were analyzed, respectively. Evaluation of the inorganic and organic content of the stones was accomplished by determining the fractions (in wt.%) of calcium phosphate and calcium oxalate minerals (via the stoichiometry of octacalcium phosphate (OCP) and whewellite (COM)/wedellite (COD)), followed by calculation of the organic fraction (based on carbon, oxygen and – less frequently – sulfur). Other elements, such as Na, Cl, K, Al, Zn, Mg, were considered as trace compounds (e.g. NaCl, KCl, Al and Zn oxides). As the stones were coated with Os for SEM the Os-content was subtracted before calculating stone compositions.

### Micro-Raman Spectrometry

Raman spectra were recorded on polished surfaces of embedded samples using a Horiba Jobin Yvon LabRAM HR (High Resolution) 800 spectrometer equipped with a Si-based CCD-detector (Peltier-cooled), an integrated Olympus BX41 optical microscope and an automatized x-y-stage at University of Mainz. Measurements were carried out with 50× long distance objective (numerical aperture 0.55) and a slit width of 100 µm, choosing laser spot sizes of ca. 2×2 µm. The excitation source was a Helium-Neon laser operated at 632.82 nm. The Rayleigh radiation was blocked using two edge filters and the scattered light was dispersed by a grating with 1800 grooves/mm. Spectra were calibrated using the 520.5 cm^−1^ band of a silicon wafer before starting measurements. All spectra were recorded twice. The wavenumber accuracy was ±0.5 cm^−1^ at a measured spectral resolution of 0.6 cm^−1^ (Full Width at Half Maximum of the Rayleigh-line).

## Results

**Figure 1:**
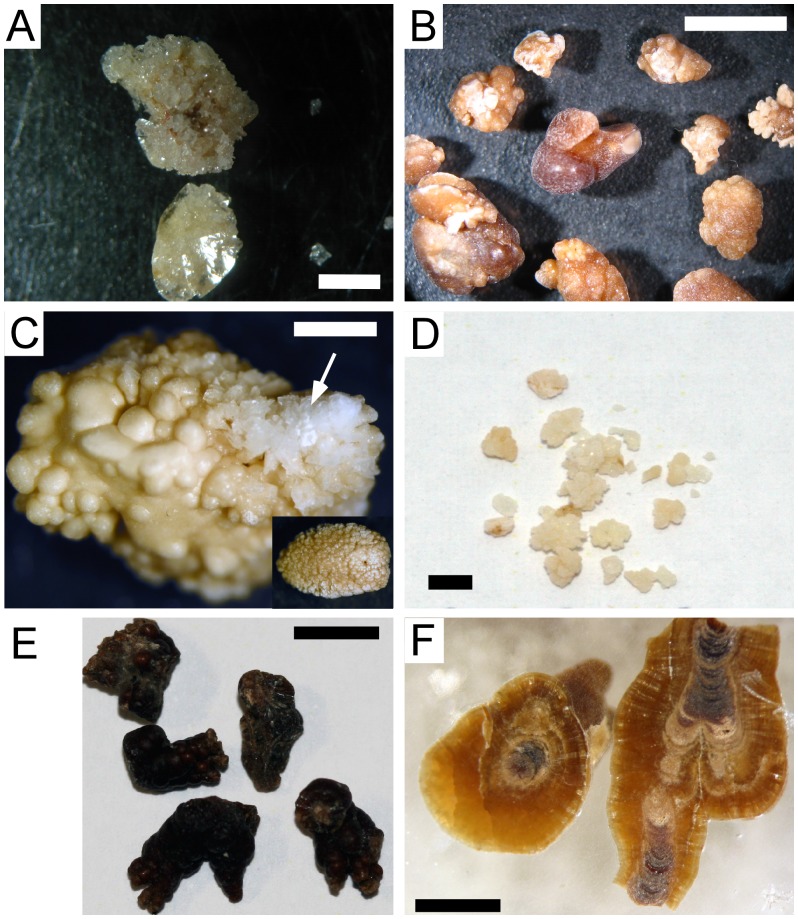
Light microscopy photographs of typical calculi from patients diagnosed with primary hyperoxaluria. Panels A and B show stones from patients diagnosed with PH I before receiving treatment (A, sample H18) and stones formed under treatment with citrate and vitamin B6 (B, sample H9). Panels C and D depict stones from an untreated PH III patient (C, sample H28) and from a patient treated with citrate (D, sample H4). Note in 1C the large very fine-grained COM region and smaller crystalline region (arrow) with bipyramidal COD crystals. The inset shows the back of this stone. Panels E and F depict typical idiopathic Ca-Ox stones for comparison consisting of COM only. Note the dark pigmentation and the characteristic core and mantle structure in the cut and polished cross-section. Scale bars A = 2 mm, B = 500 µm, C = 200 µm, D = 250 µm, E = 500 µm, F = 250 µm.

**Figure 2:**
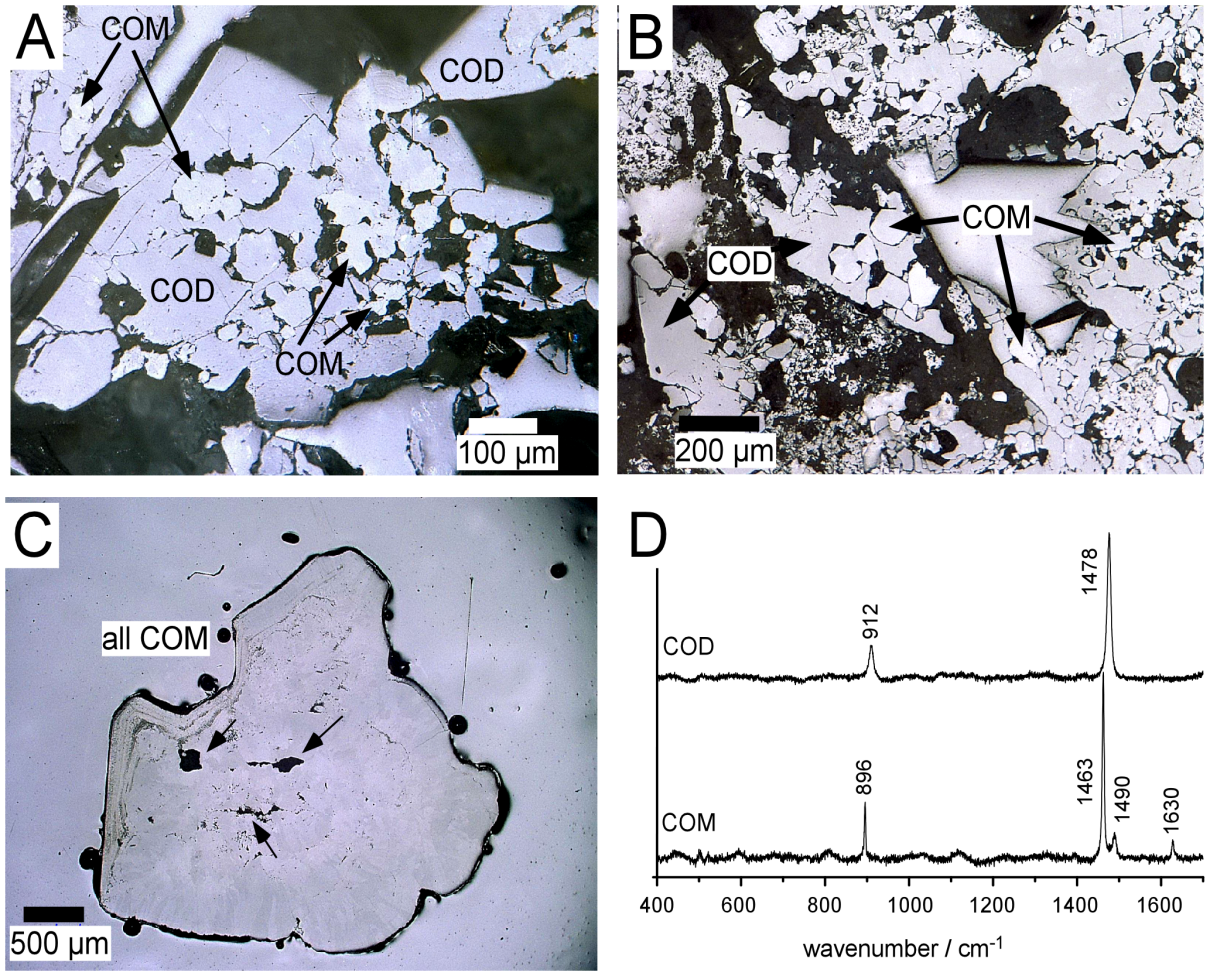
Reflected light microscopic images (polarized light) of polished sections of selected stones mounted in epoxy resin (A–C) and exemplary baseline-corrected Raman spectra (D) that identify COD and COM in the analysed stones. Stones in (A) and (B) are from patients diagnosed with PHI (A, sample H18) and PHIII (B, sample H11), but as yet untreated. The stone in C (sample H10) is from a PHI patient receiving treatment with citrate and vitamin B6 (Table 1). Note the aggregated and brittle appearance of the stones in A&B in contrast with the massive appearance of the one formed under treatment (C). Arrows in C point to small cavities in the otherwise compact stone matrix. Black features in the micrographs are holes and gaps in the mounts; the epoxy matrix is light grey. Phases were identified by Raman spectroscopy and characteristic measured spectra for COM and COD are shown in panel D. COM appears as light grey small grains in A, B and C and is clearly identified in D by the double Raman band at 1463 and 1490 cm^−1^, while COD has a single band at 1478 cm^−1^ [45]. COM = calcium oxalate monohydrate, COD = calcium oxalate dihydrate.

Calculi from patients with primary hyperoxaluria have a significantly different appearance from idiopathic CaOx stones [23], [24]. While the latter are dense and strongly pigmented calculi (Fig. 1E, F), the majority of PH stones have a light surface colour (Fig. 1A–D) and most consist of loose aggregations of different-sized crystals. Augmenting deductions from the current literature, we found that PH I and especially PH III stones are not always uniform in appearance and composition. In addition to the loose crystal aggregations, reportedly typical for PH stones, a number of calculi contained compact areas that display smooth surfaces with fine-grained growth laminations (Fig. 1C). These areas were identified as calcium oxalate monohydrate (COM; Fig. 1C) by micro-confocal Raman spectrometry. The aggregated portions of stones contain calcium oxalate dihydrate (COD) as the major component (e.g. Fig. 1C, arrow), in which the COD crystals displayed the typical bipyramidal crystal-shape (Fig. 2A, B). Ca-Oxalate Monohydrate (COM) was also present, but only as a minor phase.

Relating the stone compositions and morphologies to genetic (PH type) and clinical data showed that stones consisting partly or completely of aggregated COD were from (i) the single PH I individual who had not yet received treatment (Fig. 1A, Table 1) and (ii) PH III patients. In the PH III group, a clear discrimination of stone compositions in treated and untreated patients was not possible based on our samples. Here, both groups showed aggregated COD as well as mixtures of aggregated COD and fine-grained COM (Fig. 1C, D).

In contrast, fine-grained stones consisting almost completely of COM were formed by all other nine PH I patients already under treatment with citrate in combination with vitamin B6 or magnesium (Table 1).

A characteristic PH I concrement formed under treatment is shown in cross-section in Fig. 2C. These calculi are more compact, finer-grained and typically consist almost exclusively of COM. These features are also found in idiopathic CaOx stones, but the latter have a very distinct internal structure with an organic-rich core and crystalline mantle (Fig. 1F) and are darker in appearance (Fig. 1E).

All these characteristics are very different from the PH I stones formed under treatment which, although showing some cavities (arrows in Fig. 2C), lack the typical organic-rich core/crystalline mantle structure.

**Figure 3:**
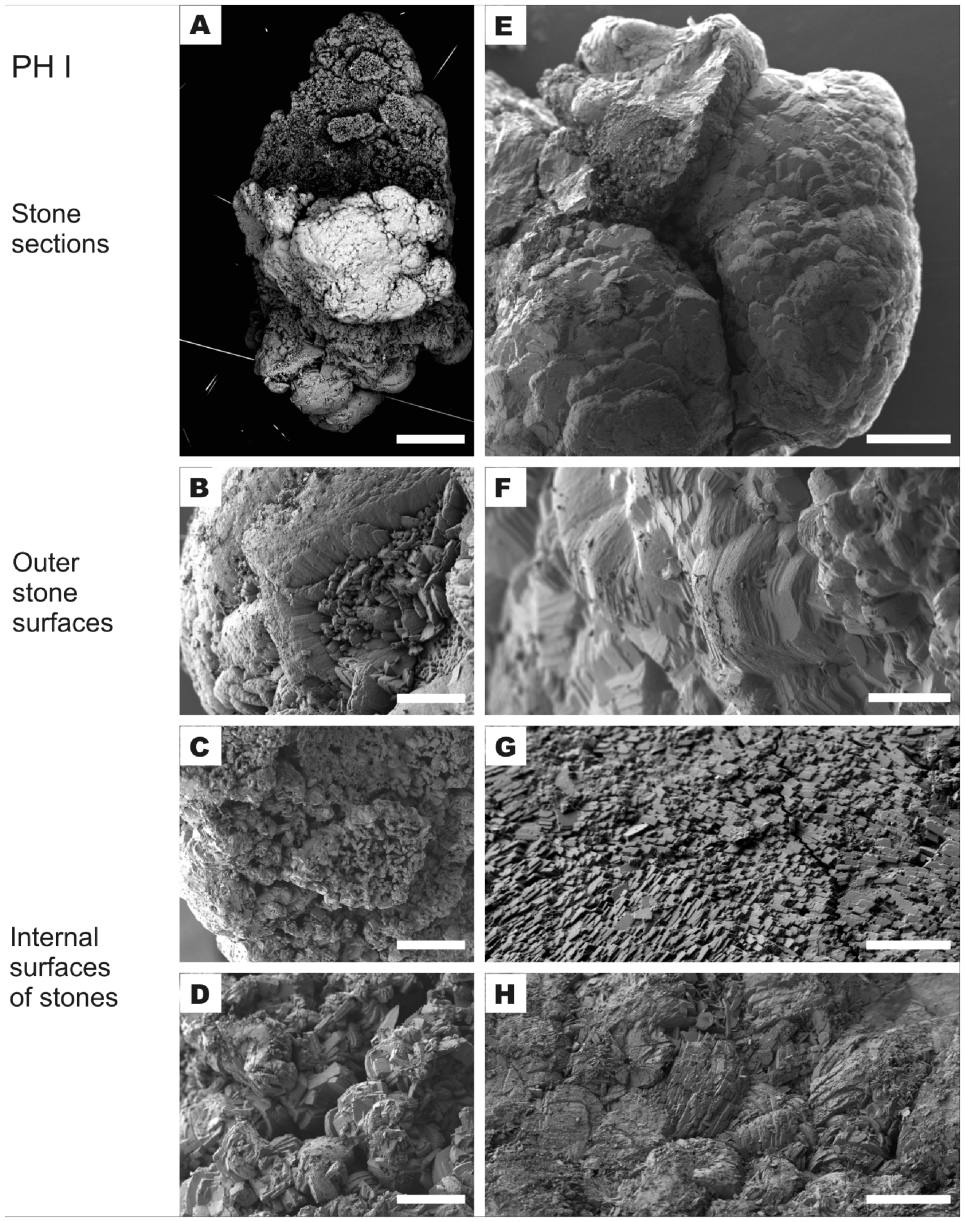
SEM micrographs of PHI stone fragments from the treatment group. Panels A–D are images of stones (samples H1, H3) showing open structured crystal aggregates. Surfaces (B) of stones and internal structures (C, D) appear to be formed by COM crystals. Panels E–H show surfaces (F) and internal structures (G, H) from more compact PHI stones (samples H6, H9, H10). Scale bars A, E: 400 µm; B, D, F: 40 µm; C: 115 µm; G: 4 µm; H: 100 µm.

Crystal morphologies of stones from treated PH I patients determined by Scanning Electron Microscopy show densely-packed aggregates of COM crystals (Fig. 3). These aggregates are composed of individual rosette-like aggregates (Fig. 3A–D, E, H) and sections of stacked crystals (Fig. 3E–H) with randomly distributed and variably-sized cavities between them, corresponding to those seen in the polished stone cross-section in Fig. 2C.

**Figure 4:**
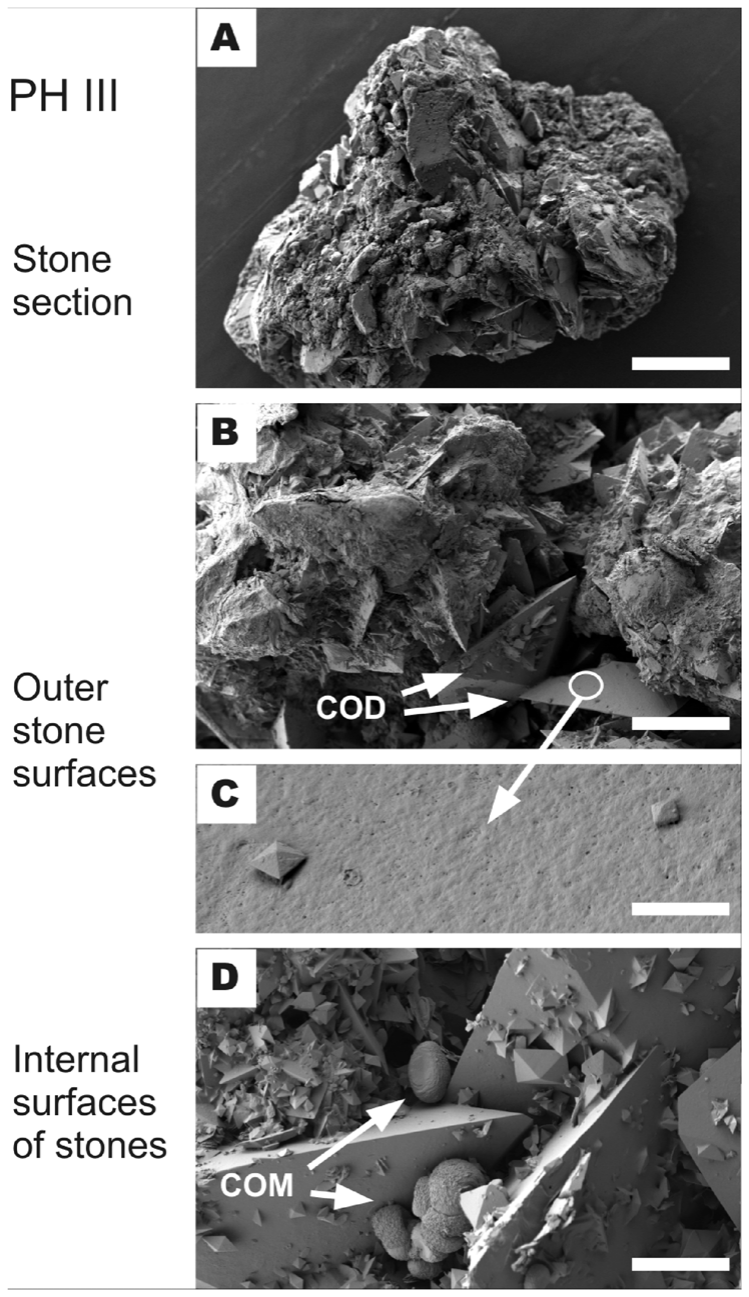
SEM micrographs of PHIII stone fragments. Panels A–D show a complete stone (A), surface characteristics (B, C) and internal structures (D). Panel C reveals a section of B showing small COD crystals (bipyramidal shapes) grown on a large uneven COD surface. The unevenness possibly originates from organic compounds coating the crystal surface. Panel D shows doughnut-shaped COM crystals in the interstitial spaces between large COD crystals. Scale bars (sample ID) A: 400 µm (H11); B: 100 µm (H13); C: 4 µm (H13); D: 14 µm (H7).

In contrast, stones that contained large amounts of COD, the PH III calculi and the one from the naïve PH I patient exhibit very different crystal morphologies (Fig. 4). Both the outer surface and the internal structure are rough (Fig. 4A, B, D) which is caused by loose aggregation of crystals and heterogeneous crystal size distributions displaying both very large (some as large as 650 µm, see Figs. 4B; 2A, B) and very small (Fig. 4C) bipyramidal COD crystals. In these stones, COM occurs in two different crystal shapes and sizes. Small (<20 µm) doughnut-shaped COM crystals are found in cavities between the large COD crystals (Fig. 4D), while sectioning and polishing of the stones exposed larger (20–150 µm) angular idiomorphic crystals (Fig. 2A, B). COM with this angular crystal habit is always included or intergrown with the COD crystals and is separated from the COD crystals by a small gap.

The surfaces of the large COD crystals facing the outside in this group of stones were found by SEM-EDX analysis to be covered by organic material (possibly composed of a mixture of proteins, tissue fragments, cells etc.), with measured organic concentrations being significantly higher than those within stone fragments (80.86 wt% ±14.04 vs. 10.80 wt% ±4.20). This result contrasts with results for stones from the treatment group which show heterogeneous organic concentrations throughout the stones, but no preferred concentration relative to their morphology (surface: 25.39 wt% ±11.48, inside: 21.32 wt% ±5.43).

## Discussion

### Effects of Treatment on Stone Composition and Texture

This study examined calcium oxalate composition and morphology in relation to the precise PH type based on mutational testing. Due to the novelty of the PH type III previous studies did not include this relevant (with regard to numbers and outcome) form and were mainly based on type I disease defined by biochemical analysis.

In addition, we tried to differentiate between calculi formed in naïve patients and those under treatment. For obvious reasons stones from patients with metabolic urolithiasis prior treatment are hard to obtain.

We are aware that our results are currently based on a very small sample number and therefore results have to be interpreted with due caution. The fact that no stones from PH II patients with an intermediate phenotype severity (regarding risk of ESRD) were available for analysis is another limitation.

Although data are limited, we feel that the findings warrant further investigations as they have diagnostic as well as therapeutic implications for the future.

Interestingly, untreated patients with PH I and PH III subtypes form stones containing considerable amounts of COD. This phase forms loose aggregates and displays a large range of crystal sizes in these calculi. In COD dominated stones, COM occurs in the interstices as well as in the form of angular crystals included and intergrown with COD. The surprising presence of COD in PH stones somehow contrasts with the findings of Daudon et al. [23], who reported a COM only composition for these stones. However, no precise clinical information with regard to treatment of the patients was given, and at the time precise genotyping of PHI-III was not available.

Only one (out of six) PH III stone analysed consisted mainly of COM, but COD was still a minor phase (Fig. 1C). Stones obtained from the PH I treatment group consisted exclusively of stones with compact matrices that were mainly composed of COM crystals with negligible COD content.

For PH III patients in our study group, we detected some increase in COM content upon treatment. However, differences were not significant, based on the fact that (i) the increase in COM content was only small and (ii) the *in situ* methods chosen for this study do not allow for representative bulk measurements. At present, it cannot be said with confidence whether the absence of a shift to COM stones relates to the different nature of the PH III subtype since the sample size in this study was still small.

The shift in phase composition of PH I stones formed under treatment to a composition almost exclusively consisting of COM may likely be related to treatment, which aims at inhibition of stone formation and growth. It is well known that citrate and magnesium inhibit nucleation and growth of CaOx stones [25]–[29]. As COD is stable in pH ranges from 5 to 7.4 [30] citrate and magnesium affect crystallization by direct interaction with the crystallizing matter rather than by increasing its solubility due to the induced change in pH [30], [31]. In addition, pyridoxine (vitamin B6) the cofactor for the defective enzyme *AGT* (PH I), markedly reduces urinary oxalate excretion in susceptible PH I patients [18], [19] resulting in an overall effect of lowering or even eliminating CaOx stone formation in response.

Thus, the compositional switch in response to treatment to COM and a dense matrix impregnated with organics form good evidence for a much reduced growth rate, besides longer residence times of the stones in the urinary tract. COD stones, in contrast, show no incorporation of organics in their matrices, thus pointing to higher growth rates in accordance with literature reports on fast nucleation and growth of COD [32].

Nevertheless, apparently higher growth rates of COD stones do not necessarily correspond to overall short renal tract residence times. Instead, our observations provide evidence for prolonged residence times of the studied COD stones in the patient’s urinary tract from phase transformation of COD to COM as outlined below.

### Phase Transformation of COD to COM

Many of the large COD crystals show inclusions of, or are intergrown with, small angular COM crystals (Fig. 2A, B). We noted that some of them were nearly completely replaced by aggregates of the small angular COM crystals, while the outer bipyramidal shape of the original large COD crystal was preserved. All of these COM crystals are in contact with COD and all show characteristic gaps between the two phases. The replacement of COD by COM, in parallel with a reduction of grain-size and increase in porosity while the external dimensions and habit of the original phase are preserved are due to a process termed pseudomorphosis which is very widespread in nature (e.g. fossilization, chemical weathering and rock metamorphism). Pseudomorphic mineral replacement is governed by solution-precipitation reactions between mineral phases and a fluid phase and is driven by the thermodynamic stabilities of the minerals involved [33].

Although COD is among the first crystals formed in the urinary tract both of healthy individuals and of stone formers [34], it is a thermodynamically metastable phase and it is well-known that COD subsequently undergoes phase transformation and is replaced by thermodynamically stable COM [35]. Although the exact rates for this transformation *in vivo* are not known, the rate of transformation is more likely to be relatively slow [36], because the dissolution of COD and precipitation of COM is surface-controlled [37]. Furthermore, numerous different proteins, cell fragments and other organic substances passivize the crystal surfaces [38].

Recent *in vitr*o studies showed that complete transformation is possible in time spans of the order of 5–75 hours [39]–[41]. This process may easily arrive at complete replacement of the dihydrate by the monohydrate phase in stones *in vivo* and depends on the residence time in the urinary tract. As the newly-formed COM crystals preserve the shape of the former COD crystal, it is possible to trace this replacement history of the stones with microanalytical methods and to discriminate the calculi in which COD was replaced by COM from those that are original contained COM stones (e.g. PH I treatment group and most idiopathic CaOx stones) [42].

### Implications for Formation Mechanisms of PH Stones

If not treated, primary hyperoxaluria results in Ca-Ox stones. Our study implies that stones formed without treatment of the patient with crystallization inhibitors originally contain a considerable fraction of COD, and this phase may even be the dominating phase in these calculi. COD crystals are found in urines of healthy individuals and of stone formers alike [43]. It is envisaged that these crystals nucleate in very large quantities and grow freely-floating in the urinary tract [44] until they reach crystal sizes that block the pathways, promoting further aggregation. Some of the COD crystals are as large as 650 µm (Fig. 2B), a size that could be retained within the child’s kidney and lead way to aggregation as the critical step for calculi formation.

Upon residence in the urinary tract the outside of the aggregated stone receives its organic coating; while COD is progressively replaced by COM within the stone (see Fig. 2A, B). This happens until finally, the complete crystal may consist of an aggregation of granular COM crystals that preserve the bipyramidal original shape of the replaced COD crystal (a so-called pseudomorph). The finding that a light-coloured CaOx stone consists mainly or even completely of COM pseudomorphs after COD could be an indication for prolonged residence times in the urinary tract. Notably, COM crystals formed via this replacement reaction have a relatively homogeneous grain-size distribution (between 20 and 150 µm), much smaller than their COD hosts. In addition, the newly formed COM aggregates are very brittle because they lack the organic coating of the large COD precursor crystals. This brittleness, for example, causes individual crystals to be lost easily upon sample polishing (exemplified by the many black voids in Figs. 2A, B).

This “downsizing effect” might be beneficial for the natural decomposition of stones and/or for supporting processes by which the smaller and non-adhesive crystals are more easily flushed out with the tubular fluid stream and excreted.

## References

[1] Hoppe B, Beck BB, Milliner DS (2009) The primary hyperoxalurias. Kidney Int 75: 1264–1271. 19225556 10.1038/ki.2009.32 PMC4577278

[2] Leumann E, Hoppe B (2001) The primary hyperoxalurias. J Am Soc Nephrol 12: 1986–1993. 11518794 10.1681/ASN.V1291986

[3] Belostotsky R, Seboun E, Idelson GH, Milliner DS, Becker-Cohen R, et al (2010) Mutations in DHDPSL are responsible for primary hyperoxaluria type III. Am J Hum Gen 87: 392–399. 10.1016/j.ajhg.2010.07.023 PMC2933339 20797690

[4] Beck BB, Baasner A, Buescher A, Habbig S, Reintjes N, et al (2013) Novel findings in patients with primary hyperoxaluria type III and implications for advanced molecular testing strategies. European Journal of Human Genetics 21: 162–172. 22781098 10.1038/ejhg.2012.139 PMC3548260

[5] Monico CG, Rossetti S, Belostotsky R, Cogal AG, Herges RM, et al (2011) Primary hyperoxaluria type III gene HOGA1 (formerly DHDPSL) as a possible risk factor for idiopathic calcium oxalate urolithiasis. Clin J Am Soc Nephrol 6: 2289–2295. 21896830 10.2215/CJN.02760311 PMC3358997

[6] Van Woerden CS, Groothoff JW, Wanders RJ, Davin JC, Wijburg FA (2003) Primary hyperoxaluria type 1 in The Netherlands: prevalence and outcome, Nephrol Dial Transplant. 18: 273–279. 10.1093/ndt/18.2.273 12543880

[7] Hoppe B, Langman C (2003) A United States survey on diagnosis, treatment and outcome of patients with primary hyperoxaluria. Pediatr Nephrol 18: 986–991. 12920626 10.1007/s00467-003-1234-x

[8] Takayama T, Nagata M, Ichiyama A, Ozono S (2005) Primary hyperoxaluria type 1 in Japan. Am J Nephrol 25: 297–302. Erratum in: Am J Nephrol 2005 25: 416. 10.1159/000086361 15961950

[9] Kopp N, Leumann E (1995) Changing pattern of primary hyperoxaluria in Switzerland. Nephrol Dial Transplant 0: 2224–2227. 10.1093/ndt/10.12.2224 8808215

[10] Hoppe B, Latta K, von Schnakenburg C, Kemper MJ, et al (2005) Primary Hyperoxaluria: the German experience. Am J Nephrol 25: 276–281. 15961947 10.1159/000086358

[11] Lieske JC, Monico CG, Holmes WS, Bergstralh EJ, Slezak JM, et al (2005) International Registry for primary hyperoxaluria and Dent’s disease. Am J Nephrol 25: 290–96. 15961949 10.1159/000086360

[12] van Woerden C, Beck B, Hutton S, Mandrile G (2010) The collaborative European cohort of primary hyperoxalurias: Clinical and genetic characterization with prediction of outcome. Pediatr Nephrol 2010 25: 1921.

[13] Kamoun A, Daudon M, Zghal A, Lasram L, BenMaiz H, et al (1997) Primary hyperoxaluria: Tunisian experience apropos of 24 pediatric cases. Nephrologie 18: 59–64. 9182235

[14] Gargah T, Khelil N, Youssef G, Karoui W, Lakhoua MR, et al (2012) Primary hyperoxaluria type 1 in Tunisian children. Saudi J Kidney Dis Transpl 23: 385–90. 22382246

[15] Hoppe B (2012) An update on primary hyperoxaluria Nat Rev Nephrol. 8: 467–475. 10.1038/nrneph.2012.113 22688746

[16] Cochat P, Liutkus A, Fargue S, Basmaison O, Ranchin B, et al (2006) Primary hyperoxaluria type 1: still challenging! Pediatr Nephrol. 21: 1075–1081. 10.1007/s00467-006-0124-4 16810517

[17] Milliner DS, Eickholt JT, Bergstralh EJ, Wilson DM, Smith LH (1994) Results of long-term treatment with orthophosphate and pyridoxine in patients with primary hyperoxaluria. N Engl J Med 331: 1553–1558. 7969325 10.1056/NEJM199412083312304

[18] Fargue S, Rumsby G, Danpure CJ (2013) Multiple mechanisms of action of pyridoxine in primary hyperoxaluria type 1. Acta Bioenerg PMID 23597595 DOI: 10.1016/j.bbadis.2013.04.010. 10.1016/j.bbadis.2013.04.010 23597595

[19] Monico CG, Rossetti S, Olson JB, Milliner DS (2005) Pyridoxine effect in type I primary hyperoxaluria is associated with the most common mutant allele. Kidney Int 67: 1704–1709. 15840016 10.1111/j.1523-1755.2005.00267.x

[20] Harambat J, Fargue S, Acquaviva C, Gagnadoux MF, Janssen F, et al (2010) Genotype-phenotype correlation in primary hyperoxaluria type 1: the p.Gly170Arg AGXT mutation is associated with a better outcome. Kidney Int 77: 443–449. 20016466 10.1038/ki.2009.435

[21] Van der Hoeven SM, van Woerden CS, Groothoof JW (2012) Primary hyperoxaluria type 1, a too often missed diagnosis and potentially treatable cause of end-stage renal disease in adults: results of the dutch cohort. Nephrol Dial Transplant 27: 3855–3862. 22844106 10.1093/ndt/gfs320

[22] Cochat P, Hulton SA, Acquaviva C, Danpure CJ, Daudon M, et al (2012) Primary hyperoxaluria Type 1: indications for screening and guidance for diagnosis and treatment. Nephrol Dial Transplant 27: 1729–1736. 22547750 10.1093/ndt/gfs078

[23] Daudon M, Estepa L, Lacour B, Jungers P (1998) Unusual morphology of calcium oxalate calculi in primary hyperoxaluria. J Nephrology 11: 51–55. 9604812

[24] Daudon M, Jungers P, Bazin D (2008) Peculiar morphology of stones in primary hyperoxaluria. New Engl J Medicine 359: 100–102. 10.1056/NEJMc0800990 18596285

[25] Fleisch H (1978) Inhibitors and prompters of stone formation. Kidney Int 13: 361–371. 351264 10.1038/ki.1978.54

[26] Hallson PC, Rose GA, Sulaiman S (1982) Magnesium reduces calcium oxalate crystal formation in human whole urine. Clin Sci (Lond) 62: 17–19. 7056030 10.1042/cs0620017

[27] Kok DJ; Papapoulos SE; Bijovet (1986) OLM (1986) Excessive crystal agglomeration with low citrate excretion in recurrent stone-formers. Lancet 1: 1056–1058. 2871335 10.1016/s0140-6736(86)91329-2

[28] Ryall RL, Harnett RM, Marshall VR (1981) The effect of urine, pyrophosphate, citrate, magnesium and glycosaminoglycans on the growth and aggregation of calcium-oxalate crystals invitro. Clinica Chimica Acta 112: 349–356. 10.1016/0009-8981(81)90458-7 6263523

[29] Tiselius HG, Berg C, Fornander AM, Nilsson MA (1993) Effects of citrate on the different phases of calcium oxalate crystallization. Scanning Microsc 7: 381–389. 8316807

[30] Chan BPH, Vincent K, Lajoie GA, Goldberg HA, Grohe B, et al (2012) On the catalysis of calcium oxalate dihydrate formation by osteopontin peptides. Colloids and Surfaces B: Biointerfaces 96: 22–28. 22503630 10.1016/j.colsurfb.2012.03.015

[31] Grohe B, O’Young J, Langdon A, Karttunen M, Goldberg HA, et al (2011) Citrate modulates calcium oxalate crystal growth by face-specific interactions. Cells Tissues Organs 194: 176–181. 21555861 10.1159/000324338

[32] Grohe B, Rogers KA, Goldberg HA, Hunter GK (2006) Crystallization kinetics of calcium oxalate hydrates studied by scanning confocal interference microscopy. J Crystal Growth 295: 148–157.

[33] Putnis A (2002) Mineral replacement reactions: from macroscopic observations to microscopic mechanisms. Min Magazine 66: 689–708.

[34] Dyer R, Nordin BEC (1967) Urinary crystals and their relation to stone formation. Nature 215: 751–752. 6059548 10.1038/215751a0

[35] Lepage L, Tawashi R (1981) Growth and characterization of calcium-oxalate dihydrate crystals (weddelite). J Pharmaceut Sci 71: 1059–1062. 10.1002/jps.2600710927 7131277

[36] Tomazic BB, Sheehan ME, Nancollas GH (1992) Influence of natural and synthetic inhibitors on the crystallization of calcium oxalate hydrates. World J Urol 10: 216–225.

[37] Tomazic BB, Nancollas GH (1980) The kinetics of dissolution of calcium-oxalate hydrates 2. The dihydrate. Investigative Urology 18, 97–101. 7410038

[38] Ryall RL, Chauvet MC, Grover PK (2005) Intracrystalline proteins and urolithiasis: a comparison of the protein content and ultrastructure of urinary calcium oxalate monohydrate and dihydrate crystals. BJU International 96: 654–663. 16104927 10.1111/j.1464-410X.2005.05701.x

[39] Breçeviç LJ, Škrtiç D, Garside J (1986) Transformation of calcium oxalate hydrates. J Crystal Growth 74: 309–408.

[40] Grohe B, Chan BPH, Sørensen ES, Lajoie G, Goldberg HA, et al (2011) Cooperation of phosphates and carboxylates controls calcium oxalate crystallization in ultrafiltered urine. Urol Res 39: 327–338. 21234554 10.1007/s00240-010-0360-8

[41] Pedraza CE, Chien YC, Mckee MD (2008) Calcium oxalate crystals in fetal bovine serum: Implications for cell culture, phagocytosis and biomineralization studies in vitro. J Cellular Biochem 103: 1379–1393. 17879965 10.1002/jcb.21515

[42] Hoppe B (2012) The enzyme 4-hydroxy-2-oxoglutarate aldolase is deficient in primary hyperoxaluria type III. Nephrol Dialysis Transplant 27: 3024–3026. 10.1093/ndt/gfs308 22851625

[43] Elliot JS, Rabinowitz IN (1980) Calcium oxalate crystalluria: Crystal size in urine. J Urol 123: 324–327. 7359628 10.1016/s0022-5347(17)55918-2

[44] Coe FL, Evan AP, Worcester EM, Lingeman JE (2010) Three pathways for human kidney stone formation. Urol Res 8: 147–160. 10.1007/s00240-010-0271-8 PMC3169174 20411383

[45] Daudon M, Protat MF, Reveillaud RJ, Jaeschke-Boyer H (1983) Infrared spectrometry and Raman microprobe in the analysis of urinary calculi. Kidney Int 23: 842–850. 6887696 10.1038/ki.1983.104

